# Mechanical properties and failure modes of CRCB specimen under impact loading

**DOI:** 10.1038/s41598-022-15985-y

**Published:** 2022-07-15

**Authors:** Wenjie Liu, Ke Yang, Litong Dou, Zhen Wei, Xiaolou Chi, Rijie Xu

**Affiliations:** 1 State Key Laboratory of Mining Response and Disaster Prevention and Control in Deep Coal Mines, Anhui University of Science and Technology, Huainan, 232001 Anhui China; 2 Institute of Energy, Hefei Comprehensive National Science Center, Hefei, 230031 Anhui China; 3 National & Local Joint Engineering Research Center of Precision Coal Mining, Anhui University of Science and Technology, Huainan, 232001 Anhui China; 4 Key Laboratory of Mining Coal Safety and Efficiently Constructed By Anhui Province and Ministry of Education, Anhui University of Science and Technology, Huainan, 232001 Anhui China

**Keywords:** Civil engineering, Coal

## Abstract

To explore the dynamic mechanical characteristics of CRCB specimens, a separated Hopkinson pressure bar (SHPB) test device combined with ultra-high-speed camera system was used to carry out the impact compression test on CRCB specimens. The stress wave propagation, dynamic stress–strain relationship, dynamic evolution of cracks, energy dissipation law and failure characteristics of the coal–rock combined body in the case of stress waves entering coal from rock were compared and analyzed. The influence of the difference between the rock and the incident bar on the propagation of stress wave gradually weakens with the increase of the impact velocity. The strength stress and peak strain of the CRCB specimens have obvious strain-rate effects. Besides, with increased impact velocity, the incident energy increases linearly, the reflected energy proportion decreases linearly and the absorbed energy proportion change approximately as a power function. Under the same stress wave, as the strength of the rock increases, the failure degree of coal gradually increases, the broken particles gradually transition from massive to powder and the rock mode changes from splitting failure to shear failure. As a result, the average particle size of broken coal blocks decreases, and the fractal dimension of CRCB specimens increases gradually. The research results provide basic research for the control of surrounding rock of roadway under dynamic pressure.

## Introduction

During mining of coal seams, the coal stratum interacts with the upper (roof) and bottom (floor) rock strata, while the remaining coal body (coal pillar) and the rock layer form a new load-bearing structure, which is referred to as a coal–rock combined body (CRCB)^1,2^. Due to excavation disturbance and strong ground pressure, the CRCB structure will inevitably experience dynamic stress waves. Because of the complicated structure of CRCB specimens, their stress wave propagation and attenuation characteristics differ from coal and rock single bodies. On the other hand, the dynamic response characteristics of the combined structure play a vital role in the stability of the roadway-surrounding rock-bearing system^3–7^. Therefore, the study of the propagation of stress waves in the CRCB specimens and their dynamic mechanical response characteristics is very topical, since it can provide a better understanding of the stability of the CRCB structure, which has great significance to control the deformation and instability of surrounding rock in dynamic pressure roadways and ensure the safe and efficient mining of mines.

There are multiple in-depth studies on the mechanical properties^8–15^, energy evolution behaviour^16–18^, deformation and failure characteristics^19–26^, constitutive models^27,28^ and destruction criteria^29–31^ of multiphase coal and rock layered composite structures via laboratory tests, theoretical analysis, numerical simulation, etc. For example, Chen et al.^2^ analysed the evolution of deformation and strength parameters of roof-coal pillar structures with different height ratios based on uniaxial compression tests of roof sandstone-coal pillar structures, which revealed the progressive failure mechanism of the coal–rock structure. Li et al.^23^ carried out impact tests on the CRCB specimens, determined their energy dissipation and crushing characteristics, and determined the influence law of the precast crack angle on their energy evolution and fractal characteristics. Gong et al.^22^ used SHPB test system to determine the impact mechanical characteristics of the CRCB under different strain rates. The test results showed that the dynamic compressive strength, dynamic peak strain, incident energy, and reflected energy of the CRCB had obvious strain rate effects. Han et al.^24^ studied the dynamic characteristics of sandstone under different cement mortar cementation thicknesses and pointed out that with the increase of cementation thickness, the failure pattern of sandstone changed from tensile spalling to splitting. In terms of mechanical constitutive equations. Liu et al.^27^ established two kinds of damage constitutive models of coal bodies by connecting damaged bodies and Newtonian body, which revealed the influence of rock on the mechanical behaviour of coal in the CRCB specimens. Based on the impact loading tests of CRCB specimens with different combination ratios. Xie and Zheng^28^ constructed a multi-parameter composite constitutive model of CRCB specimens, and the model fitting curve was in good agreement with the measured dynamic constitutive curve. Zhao et al.^29^ established an equivalent uniform model of CRCB based on the principle of equivalent strain energy in terms of failure criteria. They derived the compression-shear failure criterion considering the cohesive strength of the coal–rock interface. Yin et al.^30^ used the homogenization theory to treat the composite rock formations as an equivalent homogeneous rocks and then established a failure criterion for the layered composite rock based on the modified Lade criterion under true triaxial stress conditions. Fractal theory has been widely used in the analysis of material failure characteristics and crack propagation. For example, Maruschak et al.^32^ applied fractal theory to analyse the deformation of multi-defect materials. The research results showed that fractal dimension increased uniformly with the increase of material damage area.

The effects of coal and rock height ratio, cementation characteristics of coal and rock samples and stress loading methods on mechanical properties, deformation and failure characteristics and energy dissipation of CRCB specimens are studied in the above studies, but most of them focus on static load. The occurrence characteristics of coal seams are not only the thickness and dip angle of coal seams changing, but also the roof conditions of coal seams affected by geological action, such as scour zone and magmatic rock intrusion area, etc. The existing studies have shown that the dynamic characteristics of composite rock mass are significantly different under the influence of lithology^33^. Therefore, the author uses SHPB test system to analyze the dynamic mechanical properties of CRCB specimens under different lithologic combination conditions. in order to provide some reference for the excavation and protection of composite coal and rock engineering geological body. In order to provide some reference for the excavation and protection of complex coal and rock engineering geology rock mass.

## SHPB test of CRCB specimens

### Preparation of CRCB specimens from rock and coal samples

**Figure 1:**
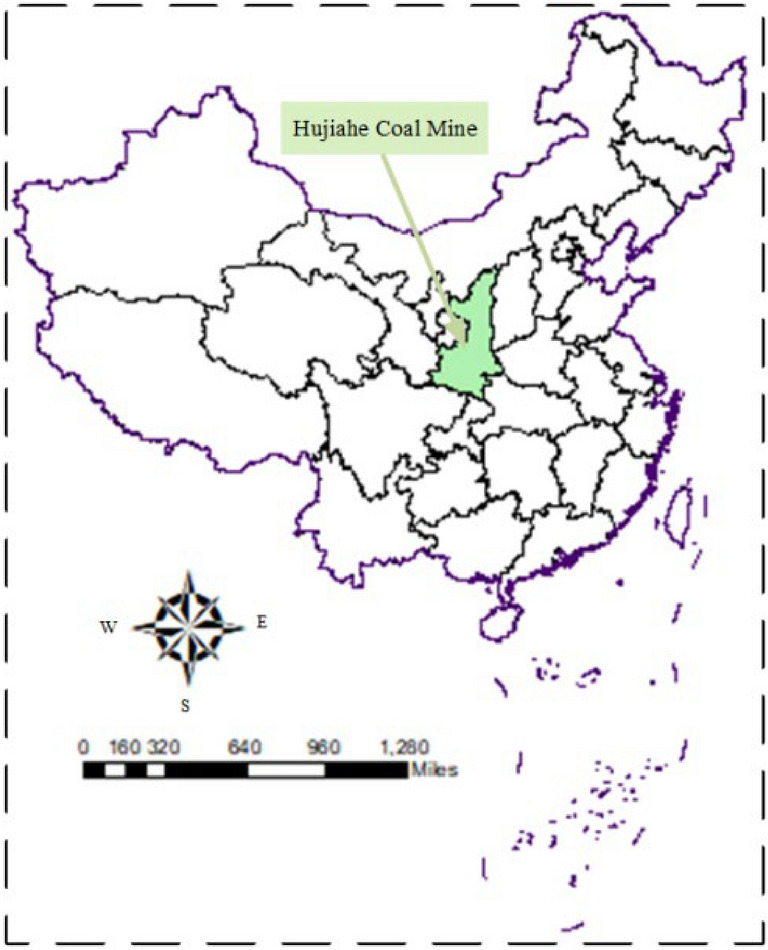
The location of Hujiahe Coal Mine (Arcgis10.8 https://www.esri.com/en-us/arcgis/products/mapping/overview).

**Figure 2:**
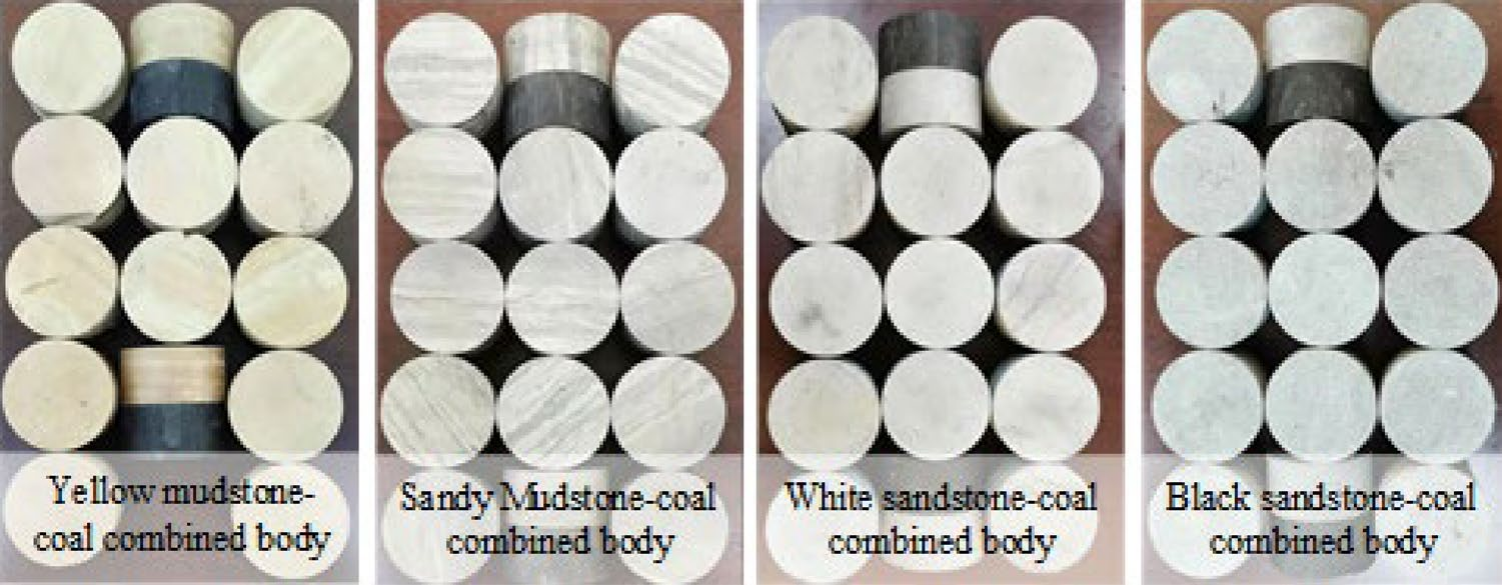
CRCB specimens prepared with four different rock sample and identical coal samples.

The coal samples required for the CRCB specimen impact tests were acquired from the 401,111 working face of the Hujiahe Coal Mine, which is located in the Binchang Mining Area, Shanxi Province of China (Fig. 1). The #4 coal seam has a high burst tendency^34^. The rock samples were collected from some rock burst mines in Huainan, Shandong, and Inner Mongolia, China. The coal and rock samples with good integrity and homogeneity were cored and cut, and processed into Ф50 mm × 25 mm cylinder samples. The rock grinding machine was used to polish the two end faces of the coal and rock samples, and the non-parallelism and non-perpendicularity of the end faces of the coal and rock samples were controlled within ± 0.02 mm. Epoxy resin is used to splice processed coal and rock samples to obtain CRCB samples. The amount of epoxy resin and bonding thickness are strictly controlled in the splicing process. Existing research results show that epoxy resin can simulate rock mechanical behavior^35^, and the mechanical characteristics of epoxy resin is the same as Yue et al. have studied^36^. According to the sample combinations, four sets of CRCB specimens were produced (Fig. 2), which comprised: (i) yellow mudstone-coal sample (MC), (ii) sandy mudstone-coal sample (SM-C), (iii) white sandstone-coal sample (WS-C), and (iv) black sandstone-coal sample (BS-C). When each specimen was processed, it was required that the size and processing accuracy of the coal and rock single body samples and the CRCB specimens met the standard ISRM requirements. In addition, the uniaxial compression specimens and Brazilian discs were prepared from rock and coal samples. The basic mechanical parameters of the coal and rock samples were determined and listed in Table 1.

**Table 1:** Mechanical parameters of coal and rock samples.

Lithology	Compressive strength/MPa	Elastic modulus/GPa	Tensile strength/MPa	Poisson's ratio	Longitudinal wave velocity/(m s^−1^)	Density/(kg m^−3^)
Yellow mudstone	8.43	1.07	0.81	0.22	2976	2124
Sandy mudstone	13.36	1.92	1.28	0.19	3276	2279
White sandstone	44.62	6.65	4.06	0.16	3846	2577
Black sandstone	65.29	7.12	5.02	0.14	3452	2718
Coal sample	13.87	2.04	1.32	0.25	2420	1481

### SHPB test system and test plan

#### Test system

**Figure 3:**
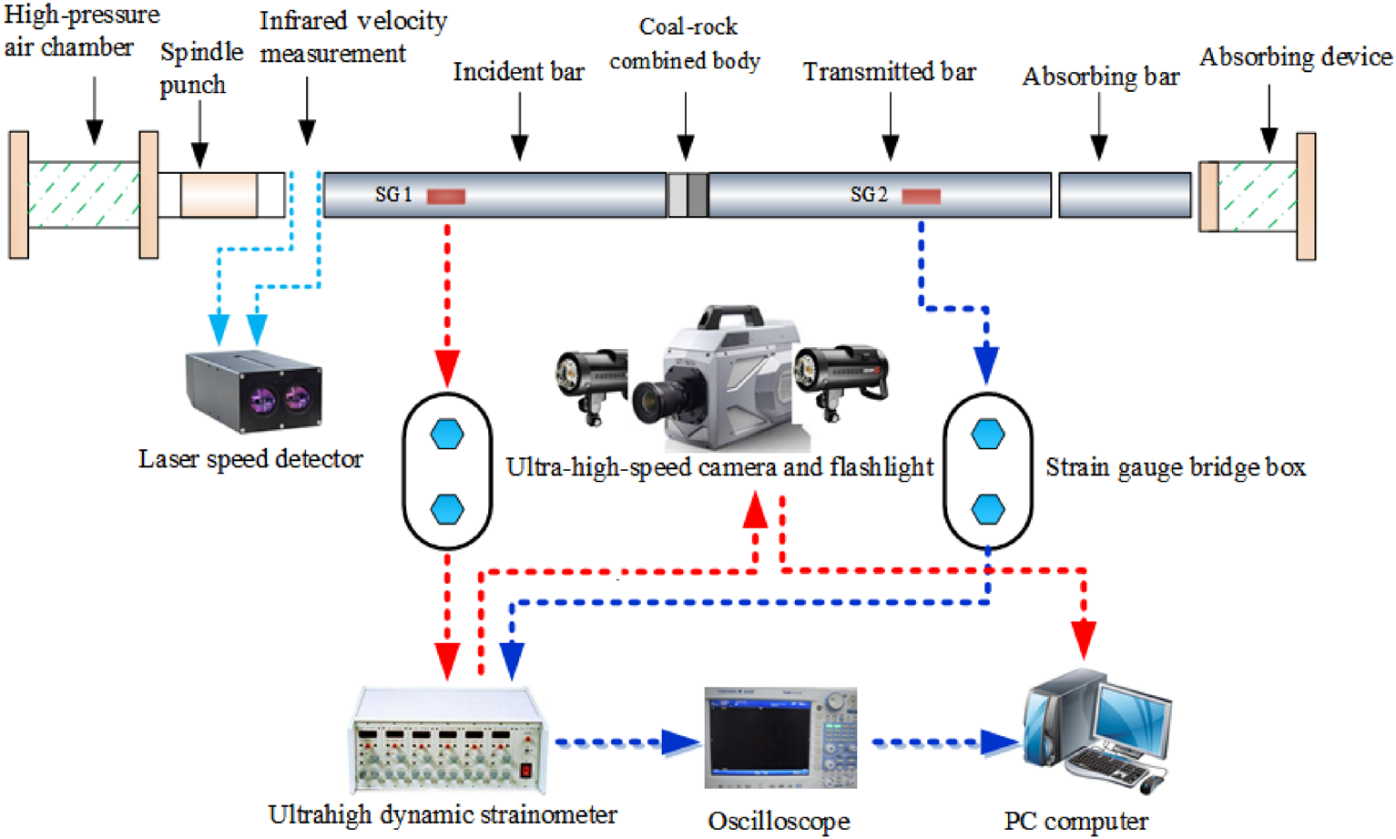
The SHPB test system.

Impact compression tests of the CRCB specimens were conducted by using the SHPB test system and ultra-high-speed camera system in the State Key Laboratory of Mining Response and Disaster Prevention and Control in Deep Coal Mines, Anhui University of Science and Technology, China. As shown in Fig. 3, the incident bar, transmission bar and bullet of SHPB test device are cylindrical steel bars with diameters of 50 mm, elastic modulus of 210 GPa and longitudinal wave velocity of 5190 m/s. During the test, impact velocity and amplitude of the incident stress waves were controlled by adjusting the nitrogen pressure inside the high-pressure chamber or changing the position of the spindle punch. Dynamic strain gauges (SG1 and SG2) and SDY2107A super-dynamic strain gauge were used to measure strain signals in the incident and transmission bars. And then strain signals were stored and displayed by Yokowaga-DL850E oscilloscope. Ultra-high-speed camera system was composed of a FASTCAM SA-Z high-speed camera and flash. Before the test, the image resolution, shooting speed, and shooting time of the high-speed camera were preset at 256 pixel × 408 pixel, 120,000 fps, and 200 μs, respectively. When the bullet hits the incident bar, the incident pulse signal will be generated, which is converted into voltage signal by the dynamic strain gauges (SG1) at the front of the incident bar. The voltage signal will make the SDY2107A super-dynamic strain gauge to trigger the operation of high-speed camera and flash. The system can clearly capture the whole process of sample failure and meet the test requirements.

#### Test plan

The impact test design of CRCB specimens envisaged that during the impact loading process, the stress wave entered the coal component from the rock component. Table 1 shows that the physical and mechanical parameters of the selected coal and rock samples were quite different. The uniaxial compressive strength values and elastic moduli of the rocks ranged from 8 to 70 MPa and 1 to 8 GPa, respectively. Therefore, it was crucial to select an appropriate impact air pressure and impact velocity. Therefore, impact trials were performed on the coal and rock samples before the test. The trial results show that the mudstone and coal samples with lower strength were more intensively fractured under the action of low impact pressure. When the impact pressure exceeded 0.6 MPa, macroscopic crack initiation and expansion began to occur in the sandstone. Therefore, the impact tests were performed under five impact air pressures of 0.4, 0.5, 0.6, 0.7, and 0.8 MPa, with more than three specimens tested at each impact air pressure. Before the tests, a thin layer of Vaseline was applied to the contact between the specimen and the bar to reduce the friction effect on the end surface.

#### Dynamic stress balance verification

**Figure 4:**
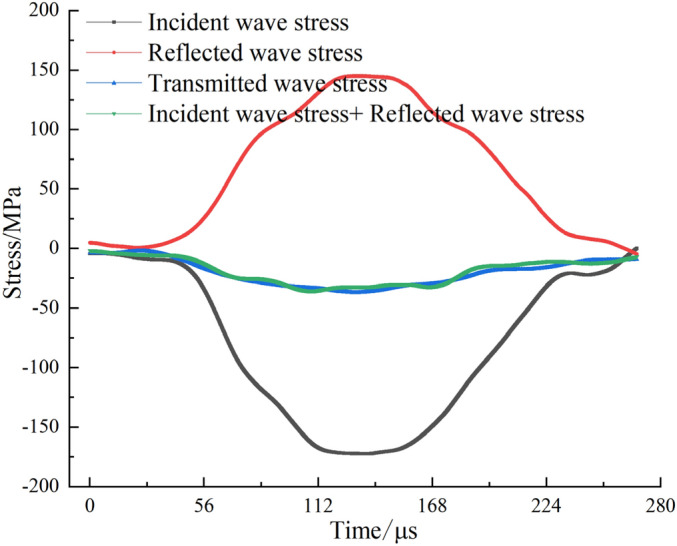
Verification of dynamic stress balance for a typical specimen.

To ensure the reliability of the test results, the stress balance of CRCB specimen was verified. Figure 4 shows the stress evolution in a CRCB specimen subjected to impact compression loading, where the sum of the incident stress and the reflected stress is approximately equal to the transmission stress, indicating that the stress balance conditions is satisfied. Other group tests also satisfy stress balance conditions, which will not be described here.

### Test results and analysis

#### Stress wave propagation characteristics

**Figure 5:**
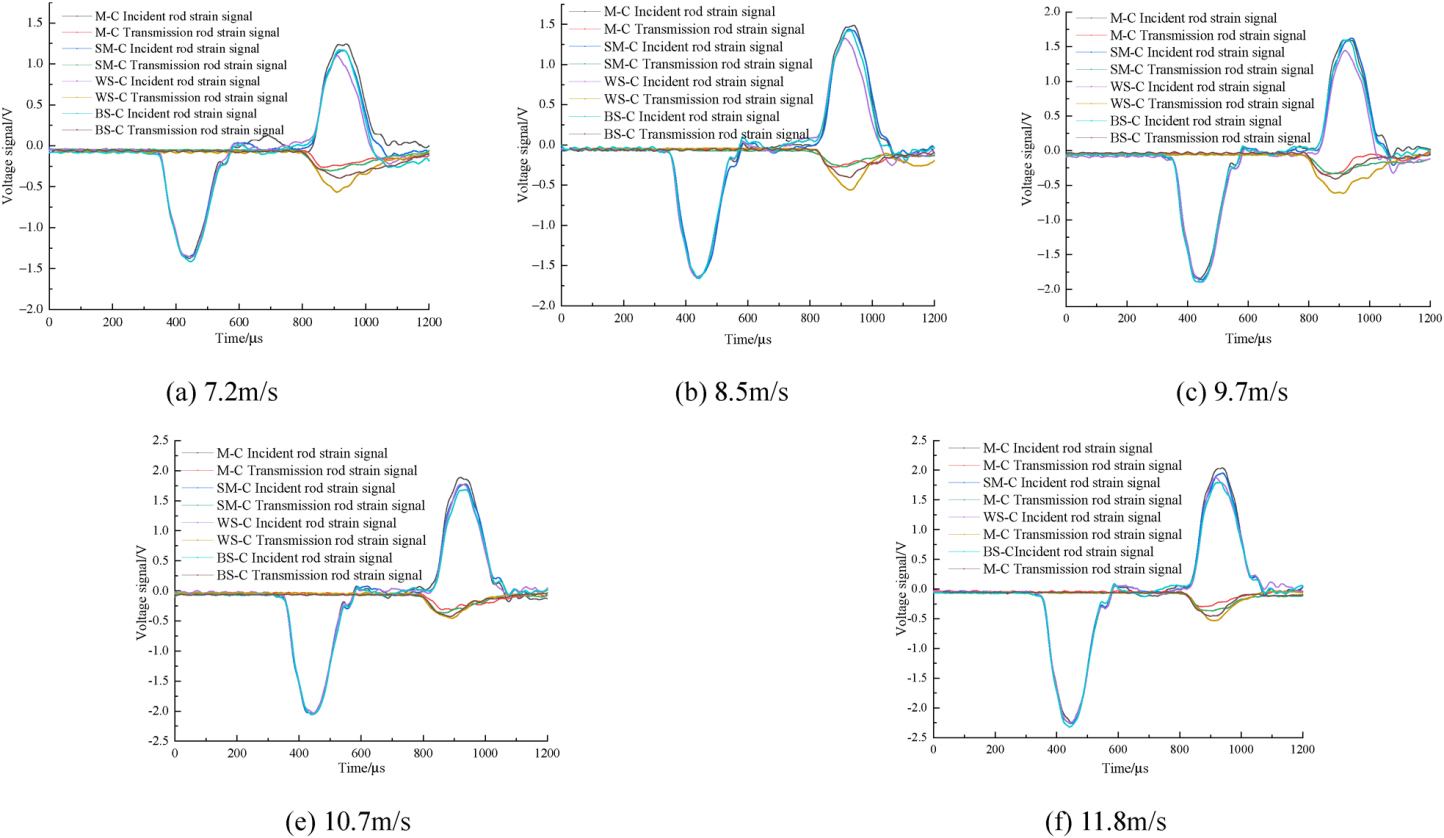
The waveforms in CRCB specimens at different impact velocities.

Comparing the stress wave characteristics of CRCB specimens under different impact velocities (Fig. 5), it can be obtained that: as the impact velocity is increased, the amplitudes of the incident wave and reflected wave of the CRCB specimens gradually increase. Under the same impact velocity, the incident waves of each group of CRCB specimens were the same. However, as the wave impedance of rock increased, the amplitude of the transmitted wave gradually increased, while the reflected wave amplitude decreased. Such a difference was more obvious when the impact velocities is range from 7 to 10 m/s, but less pronounced at high impact velocities (10–12 m/s). From the stress wave propagation characteristics, it can be concluded that the greater of the rock wave impedance is, the better matching effect between the CRCB specimens and the incident bar are. More stress waves will propagate to the transmission bar through the CRCB specimen when the incident wave propagates to the interface between CRCB specimen and the incident bar. Therefore, under the same impact velocity, the amplitude of the WS–C specimens transmission wave should be the largest, while the amplitude of the M–C specimens transmission wave should be the smallest. However, with the impact velocity increases, the impact of the difference between the wave impedance of CRCB specimens and the wave impedance of incident bar on the stress wave propagation gradually decreases, resulting in similar amplitude of transmitted waves.

#### Characteristics of the dynamic stress–strain curve

Based on the assumption of one-dimensional stress wave and stress–strain uniformity, according to the incident wave *ε*_I_(*t*) and reflected wave *ε*_R_(*t*) measured by the strain gauge on the incident bar and the transmitted wave *ε*_T_(*t*) measured by the strain gauge on the transmission bar, the stress and strain of the CRCB specimens can be calculated^37–39^:

1 $$\sigma (t) = \frac{{A_{0} }}{{2A_{S} }}E_{0} \left[ {\varepsilon_{I} (t) + \varepsilon_{R} (t) + \varepsilon_{T} (t)} \right],$$

2 $$\varepsilon (t) = \frac{{C_{0} }}{{L_{S} }}\int {\left[ {\varepsilon_{I} (t) - \varepsilon_{R} (t) - \varepsilon_{T} (t)} \right]} dt,$$

where A_0_ is the cross-sectional area of the bar, mm^2^; E_0_ is the elastic modulus of the pressure bar, GPa; C_0_ is the longitudinal wave velocity of the member, m/s; L_s_ is the length of sample, mm; As is the cross-sectional area of the CRCB specimens, mm^2^.

**Figure 6:**
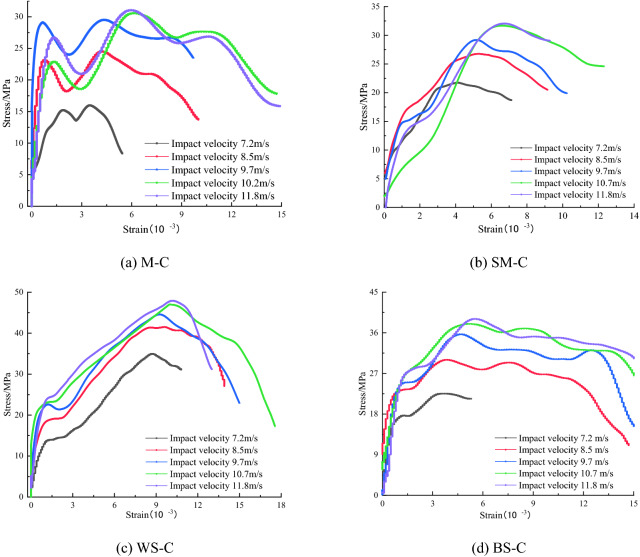
Equivalent stress–strain curves of CRCB specimens.

Figure 6 shows that the stress–strain curves within the same CRCB group were similar, while the shapes of the stress–strain curves in different groups were quite different. That is to say the propagation and attenuation patterns of stress waves in the same type of CRCB specimens were similar. The CRCB specimens had no pronounced compression and compaction stages. In addition, when the coal and rock in the CRCB specimen had large differences in mechanical properties, the stress–strain curve mostly presented a "bimodal" distribution before the dynamic stress–strain curve reached strength (σ_II_).

**Figure 7:**
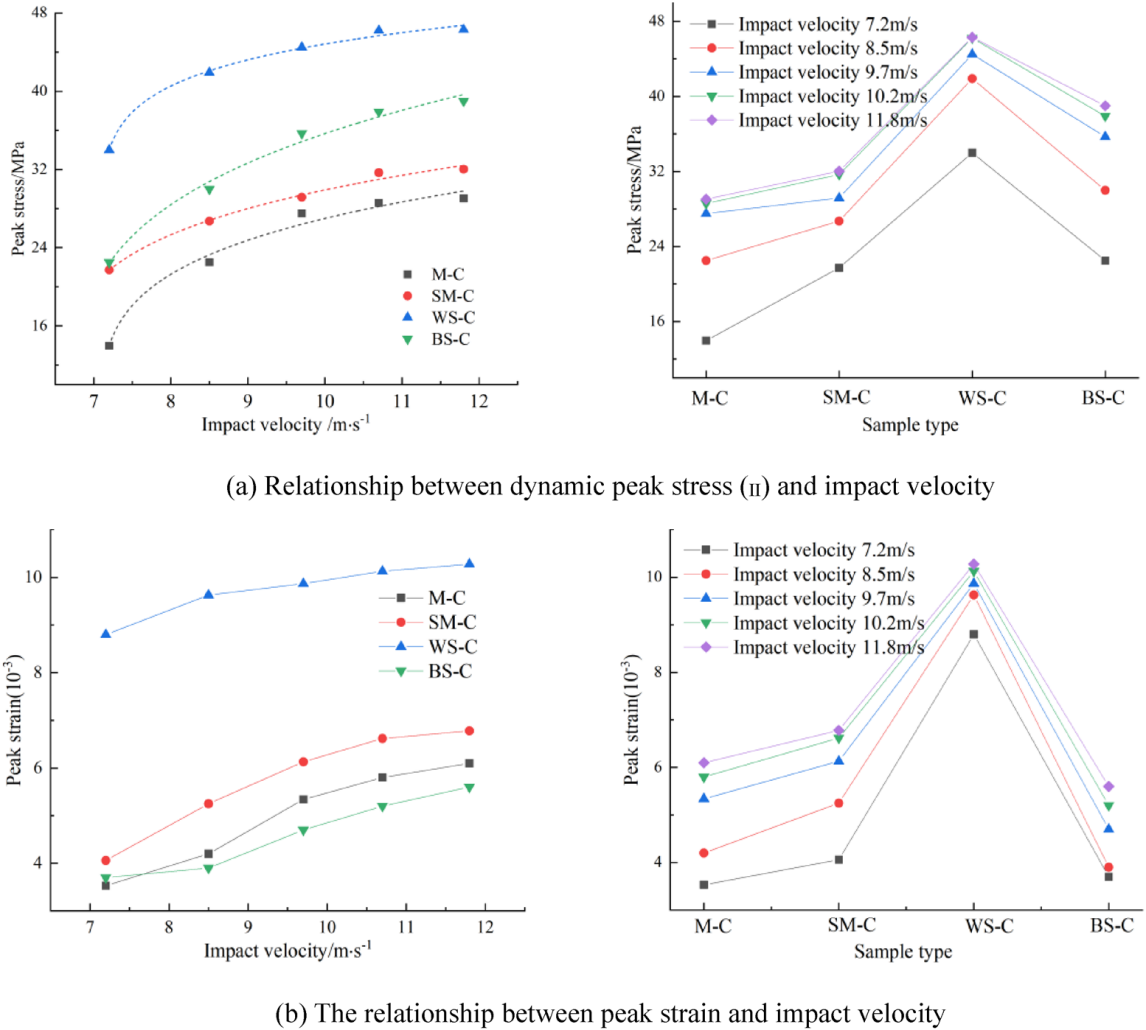
The dynamic strength and peak strain versus impact velocity for different CRCB specimens.

The stress–strain curve of the CRCB specimens possessed obvious nonlinear characteristics before it reached the first dynamic peak stress (σ_I_). With an increase in impact velocity, σ_I_ showed no obvious regular changes. At low impact velocity, σ_I_ was significantly smaller than at high impact velocity. As the impact velocity increased, the strength of CRCB specimen (σ_II_) exhibited a significant strain rate effect. Figure 7a shows that σ_II_ increased rapidly under low impact velocity and then slowly, approximately a power function, with the impact velocity increase. The equation y = *a*(*x *− *b*)^c^ was used for its best fitting, and the fitting parameters *a*, *b*, and *c* are listed in Table 2.

**Table 2:** Fitting parameters.

Sample type	a	b	c	R^2^
M-C	21.66	7.08	0.20	0.968
SM-C	23.57	6.54	0.19	0.981
WS-C	40.82	7.07	0.08	0.989
BS-C	26.71	6.71	0.24	0.978

As shown in Fig. 7b, with an increase in the strength and elastic modulus of rock, the strength and peak strain of CRCB specimens increased firstly and then decreased. Compared to WS-C specimens, the strength and peak strain of BS-C specimens were significantly smaller. The dynamic stress–strain curve of WS-C specimens increased tortuously before reaching the strength (σ_II_). Compared with that of WS-C specimens, the stress–strain curves of MC, SM-C, and BS-C specimens were approximately straight lines before reaching the strength (σ_II_), the slopes of which did not change greatly with the impact velocity. After the stress of the CRCB specimens reached the strength (σ_II_), the dynamic stress–strain curve underwent several "ups and downs". This indicates that the strain-hardening characteristic of the CRCB specimens were remarkable, and the plastic deformation was enhanced.

#### Analysis of energy dissipation characteristics

Energy accumulation, release and dissipation occur in the process of deformation and failure of CRCB specimens. It is of great significance to study the energy dissipation law of CRCB specimens under impact loading for improving the anti-impact characteristics of surrounding rock bearing structure of roadway under coal and rock composite engineering. Assumed that there is no heat exchange between the CRCB specimens and the surrounding environment during the test, and acoustic emission energy and electromagnetic radiation energy are ignored. The energy carried by stress wave can be calculated^40^:

3 $$W_{I} = A_{0} E_{0} C_{0} \int {\varepsilon_{I}^{2} } (t)dt,$$

4 $$W_{R} = A_{0} E_{0} C_{0} \int {\varepsilon_{R}^{2} } (t)dt,$$

5 $$W_{T} = A_{0} E_{0} C_{0} \int {\varepsilon_{T}^{2} } (t)dt,$$

where W_*I*_, W_*R*_ and W_*T*_ represent the energy carried by the incident wave, reflected wave and transmitted wave respectively.

According to the principle of energy conservation, ignoring the energy loss caused by friction between the pressure bar and the CRCB specimens in the process of stress wave propagation, the energy absorbed by the CRCB specimens (W_A_) under impact can be obtained:

6 $$W_{A} = W_{I} - W_{R} - W_{T} .$$

In order to analyze the law of energy propagation and dissipation of CRCB specimens under dynamic loading, the ratio of absorbed energy to incident energy of CRCB specimens is defined as absorbed energy ratio (λ), and the ratio of reflected energy to incident energy is defined as reflected energy ratio (β).

7 $$\lambda = \frac{{W_{A} }}{{W_{I} }},$$

8 $$\beta = \frac{{W_{R} }}{{W_{I} }},$$

where λ is absorbed energy ratio, β is reflected energy ratio.

**Figure 8:**
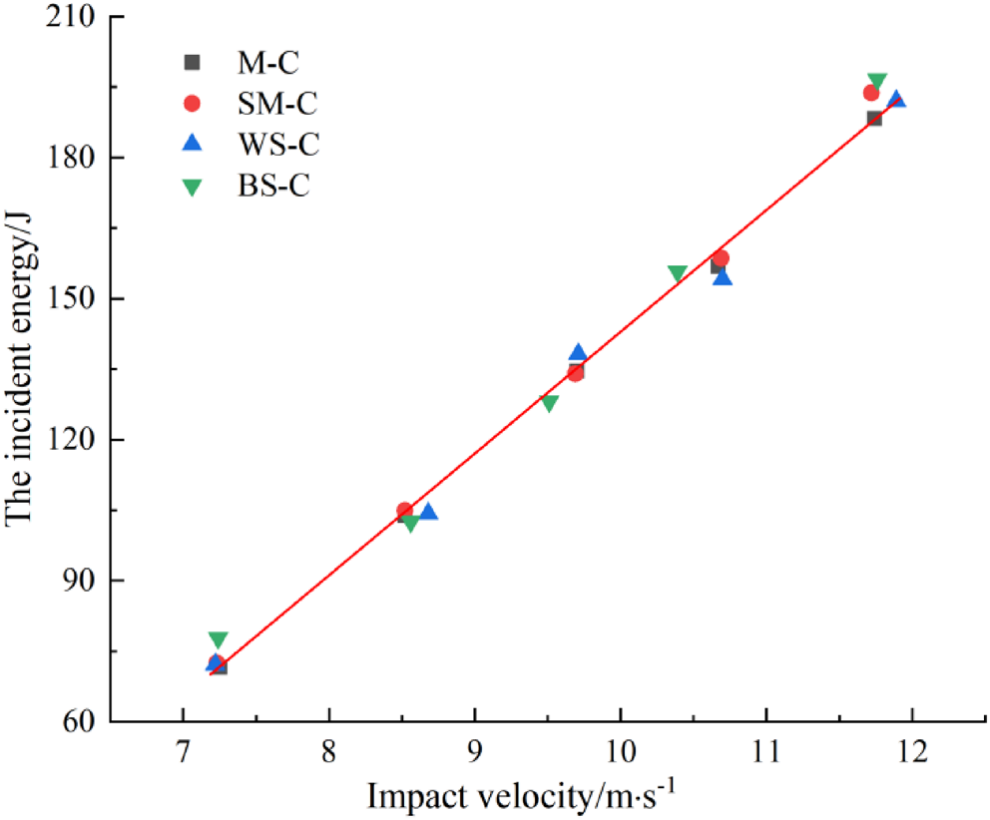
The relationship between impact velocity and incident energy.

**Figure 9:**
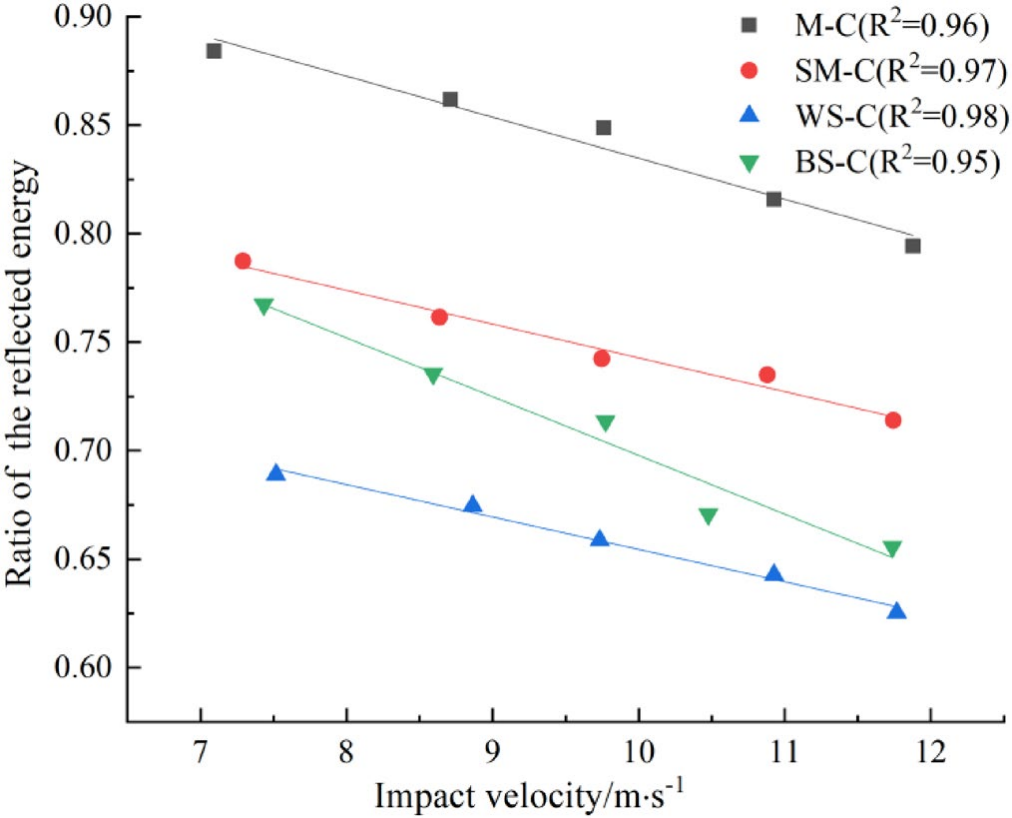
The relationship between impact velocity and ratio of the reflected energy.

**Figure 10:**
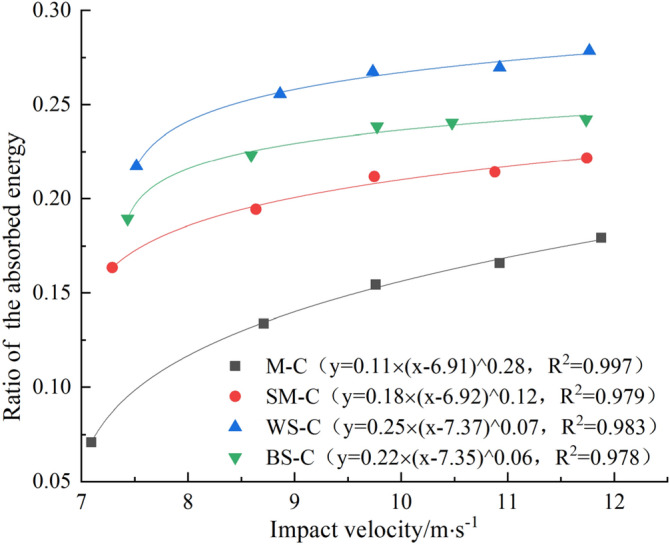
The relationship between impact velocity and ratio of the absorbed energy.

The incident energy, reflected energy, transmitted energy and absorbed energy of CRCB specimen under different impact velocities can be calculated by Eqs. (3–8). The relationship between the incident energy, the absorbed energy ratio, the reflected energy and impact velocity can be obtained by analysis, as shown in Figs. 8, 9, 10. Figures 8 and 9 shows that with the increase of impact velocity, the incident energy is independent of CRCB specimens type and increases approximately linearly. The ratio of reflected energy decreases linearly with the increase of impact velocity in the same group of CRCB specimens. Combined with the analysis of stress wave propagation characteristics, it is easy to understand that under the action of the same impact velocity, the better the impedance matching effect of rock and incident bar wave is, the more energy will be transmitted to the CRCB specimen with incident wave, and the proportion of reflected energy will decrease. Therefore, compared with other groups, the β of WS-C specimens is relatively minimum. On the other hand, with the increase of impact velocity, the effect of impedance matching between rock and incident bar wave is gradually weakened, and more incident energy will be transmitted to CRCB specimen, and the ratio of reflected energy will gradually decrease.

As can be seen from Fig. 10, the ratio of absorbed energy increases with the increase of impact velocity, but the growth rate tends to decrease, approximately presenting a power function growth. The analysis shows that under the same impact velocity, when the rock strength is low, the energy storage limit of CRCB specimen is relatively low, and less energy is required for deformation and failure.

With the increase of impact velocity, the degree of breakage of CRCB specimens gradually increases, and the energy absorbed by the specimens also increases. However, when the impact velocity is greater than a certain value, on the premise of not changing boundary conditions, the fragmentation degree of the CRCB specimens tends to be stable, and the energy absorbed during the failure of the CRCB specimens increases slowly. The proportion of absorbed energy gradually flattens out. It is worth noting that although the strength of black sandstone is greater than that of white sandstone, the *λ* of WS-C specimens is greater than that of BS-C specimens. This phenomenon will be further analyzed in combination with the fracture characteristics of the CRCB specimens.

#### Dynamic evolution characteristics of cracks

The failure mode of the CRCB specimens reflects its ability to resist impact damage. High-speed camera was used to capture the fracture process in the CRCB specimens, the dynamic evolution process of cracks in the CRCB specimens was obtained, as shown in Table 3. According to the author's existing research^11^, when the CRCB specimens are deformed and destroyed, the strength of coal or rock with large elastic modulus and small Poisson's ratios are weakened at interface. In contrast, the strength of coal and rock with small elastic modulus and large Poisson's ratios are strengthened. In the whole dynamic loading process, the axial compressive stress, elastic modulus of rock and Poisson's ratio are time-varying quantities, and are affected by impact velocity. However, the relationship between the elastic modulus and poisson's ratio of coal and rock remains unchanged. In other words, the change of axial compressive stress, elastic modulus and Poisson's ratio of coal and rock only has effect on the degree of weakening or strengthening of coal or rock strength at the interface. The lateral constraint stress may be not as pronounced as the real stress effect. However, theoretically it exists and directly affects the initiation, expansion, and arrest of cracks at the coal and rock interface.

**Table 3:** Deformation and failure process of CRCB specimens.

Impact velocity/m s^−1^	Deformation and failure process	
	Macroscopic main crack initiation	Macroscopic crack propagation
**Yellow mudstone-coal sample**		
7.2		
8.5		
9.7		
10.7		
11.8		
**Fracture pattern** (1) Under the action of impact load, most cracking and failure of the M-C sample occurred in the yellow mudstone far away from the coal–rock interface. Additionally, the number of cracks in the yellow mudstone is larger than in the coal body at the initial loading stage (2) Under the continuous action of the stress wave, when the stress at the crack tip exceeds the strength of the weakened coal sample, part of the cracks in the yellow mudstone at the interface will develop across the coal and rock interface to the coal body, inducing the overall failure of the M-C specimens (3) The coal samples show tensile failure, while compression-shear fractures dominate the yellow mudstone (4) With an increase in the impact velocity, the cracks develop intricately in the specimens. The M-C specimens is split by cracks, and the degree of fragmentation gradually increases. The volume of broken blocks gradually decreases, and the number of fragments increases. Compared with the coal samples, yellow mudstone is damaged more severely and has a higher degree of fragmentation (5) It can be seen from the initiation, propagation, and arrest behaviour of the surface cracks in M-C specimens that the strength of sandstone far away from the interface is less than the strength of sandstone at the interface		
**Sandy mudstone-coal sample**		
7.2		
8.5		
9.7		
10.7		
11.8		
**Fracture pattern** (1) The initiation of macroscopic cracks in SM-C specimens is not concentrated in a certain component but appears randomly in the SM-C specimens (2) Under the action of stress wave of dynamic loading, the main cracks of SM-C specimens are mostly vertical (stress loading direction) cracks. When the main crack crosses the coal and rock interface, the direction of crack propagation does not change (3) As the impact velocity increases, the coal and rock samples of the SM-C specimens become more broken. Both the coal and rock components mainly experience tensile failure		
**White sandstone-coal sample**		
7.2		
8.5		
9.7		
10.7		
11.8		
**Fracture pattern** (1) At the initial stage of impact loading, the macroscopic cracks in the WS-C specimen are mainly concentrated in the coal. The coal body far away from the interface is the first to swell and fracture. While the white sandstone has no obvious initiation of macroscopic cracks and showing good integrity (2) When the coal cracks expand to the coal and rock interface, the crack propagation path is blocked, and thus, coal cracks cannot penetrate the white sandstone. However, with the increase of the impact velocity, according to Griffith strength theory, when the stress at the crack tip is greater than the strength of the white sandstone, the white sandstone begins to crack and fail. The main crack in white sandstone has a large angle with the loading direction and the shear failure surface of the white sandstone increases with the increase of the impact velocity. It should be noted that at the impact velocities of 7.2 and 8.5 m/s, the white sandstone is damaged after multiple collisions with the bars, and cracks initiate in the sandstone edge (3) At low impact velocity, the white sandstone is split into large rock blocks by cracks. With an increase in the impact velocity, the degree of white sandstone fragmentation gradually increases, changing from large blocks to small ones. The coal sample also becomes more fragmented, and the size of the broken body transitions from granular to powdery		
**Black sandstone-coal sample**		
7.2		
8.5		
9.7		
10.7		
11.8		
**Fracture pattern** (1) When the impact velocity is less than 9.7 m/s, the initiation of macroscopic cracks in the BS-C specimens is mainly concentrated in the coal sample, while the rock samples has no macroscopic cracks. When the impact velocity is greater than 10.7 m/s, the black sandstone has a major crack propagation with a large angle to the loading direction, and the rock sample shows a single inclined plane shear failure (2) Under a low impact rate, the damage to the rock sample is small, and the integrity is good. However, small cracks inside the coal sample are fully developed, and the coal damaged relatively severe. The crack expansion and development are complicated, and the coal sample is broken into granular shaped particles. As the impact velocity increases, the sandstone begins to break and is divided into large pieces of rock by the cracks, and the coal sample fragments gradually turn powdery		

The green line represents coal cracks, and the black line represents rock cracks.

From the characteristics of crack propagation in the CRCB specimen, it can be seen that the macro-cracking mostly occurs at the coal or rock end far away from the coal and rock interface. When the cracks develop to the coal and rock interface, the crack expansion is blocked. However, as the impact velocity increases, when the stress at the tip of the crack is greater than the weakened strength of the coal or rock, the crack will continue to develop and pass through the coal and rock interface. Under the same impact velocity, the initiation and propagation of cracks in the BS-C and WS-C specimens significantly differ from those of the M-C and SM-C specimens, which fail by bulging and splitting, the coal is split into blocks by cracks. In contrast, coal samples in the BS-C and WS-C specimens have fully developed micro-cracks and the broken body of coal samples is granular and powdery. The absorbed energy of the CRCB specimens is mostly spent on the initiation and expansion of cracks^41^. However, it is difficult to analyse the difference in the degree of fragmentation and obtain the energy dissipation and dynamic characteristics of the CRCB specimens quantitatively under different lithologies, only from the perspective of the development of cracks on the surface of the CRCB specimens. From the perspective of crack propagation, it is difficult to quantitatively analyze the crushing degree of specimens, obtain the energy dissipation and dynamic characteristics of CRCB specimens. Therefore, it is necessary to analyse the crushing characteristics of the CRCB specimens.

### Analysis of CRCB specimens crushing characteristics

After impact tests is completed, the coal and rock broken block were collected to obtain the damage patterns of the CRCB specimen under different impact velocity, as shown in Table 4. With an increase in the impact air velocity, the fragmentation degree of CRCB specimen gradually intensified, the volume of the broken block gradually decreased. The broken block has obvious classification characteristics.

**Table 4:** Failure modes of CRCB specimens.

Impact velocity m/s	Type of coal–rock combined body (CRCB)			
	M-C	SM-C	WS-C	BS-C
7.2				
8.5				
9.7				
10.7				
11.8				

To further quantify and analyse the energy dissipation characteristics of each component of the CRCB specimens, standard sieves with sizes of 25, 20, 16, 10, 5, and 2.5 mm were selected to screen and weigh the coal and rock fragments. In order to quantitatively compare the fragmentation size of CRCB specimens, the average particle size of fragmentation(D_S_) is used to represent the fragmentation degree of CRCB specimens.

9 $$D_{s} = \frac{{\sum {\beta_{i} D_{i} } }}{{\sum {\beta_{i} } }},$$

where *D*_*i*_ is the mesh size, β_*i*_ is the mass percentage of coal and rock fragments for the mesh size of D_*i*_.

The average particle size of fragmentation D_S_ can be used to compare the crushing degree of coal and rocks in a simple and intuitive way, but it can not directly reflect the distribution characteristics of the particle size of broken coal or rocks. In other words, if the broken CRCB specimen have the same D_S_ does not mean that the mass of fragments on each sieves is the same, so the distribution characteristics of fragmentation can not be truly quantified. The research results of many scholars show that the fragmentation of rock has fractal characteristics^42,43^. The fractal dimension (D) of coal and rock fragment can reflect the distribution characteristics of coal and rock fragments intuitively and quantitatively.

10 $$D = 3 - \delta ,$$

11 $$\delta = \frac{{\lg (M_{{L_{eq} }} /M)}}{{\lg L_{eq} }},$$

where *M*_Leq_ is the mass of the fragments corresponding to the equivalent side length *L*_eq_, *M* is the mass of the fragments in the calculated size, and *D* is the fractal dimension of the fragment. *δ* is the *M*_Leq_/*M *− *L*_eq_ slope value in double logarithmic coordinates, and *M*_Leq_/*M* is the cumulative percentage content of fragments whose equivalent side length is less than *L*_eq_.

**Figure 11:**
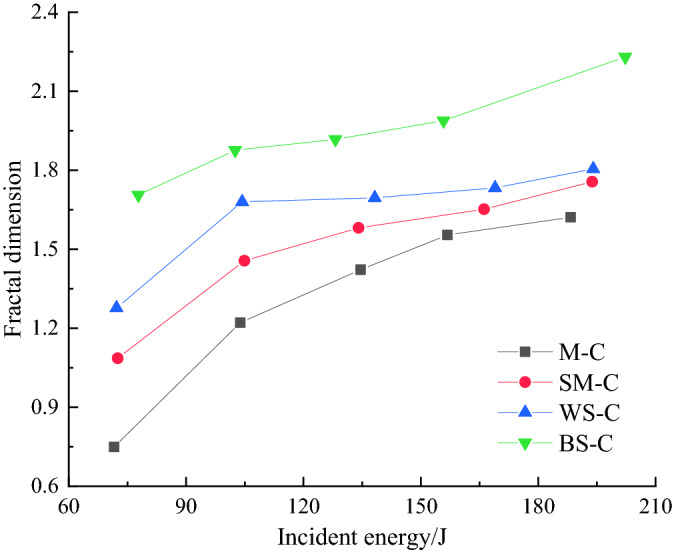
Relationship between the fractal dimension and the incident energy of CRCB specimens.

From the change law in the fractal dimension of the CRCB specimens with the incident energy depicted in Fig. 11, the fractal dimension of the CRCB specimens increased and the increment rate had a decreasing trend, with the increase of incident energy. It is worth noting that, theoretically, under the same incident energy, the lower the rock strength is, the higher the fragmentation degree of CRCB specimens should be. The more broken blocks of CRCB specimens there are, and the larger the fractal dimension is. However, under the same incident energy, the fractal dimension of the BS-C specimens was the largest, followed by WS-C and SM-C, while that of M-C specimens was the smallest. The rock samples of the BS-C specimens had good integrity and a low degree of fragmentation at an impact velocity of 10.7 m/s, according to its failure characteristics listed in Table 4.

**Figure 12:**
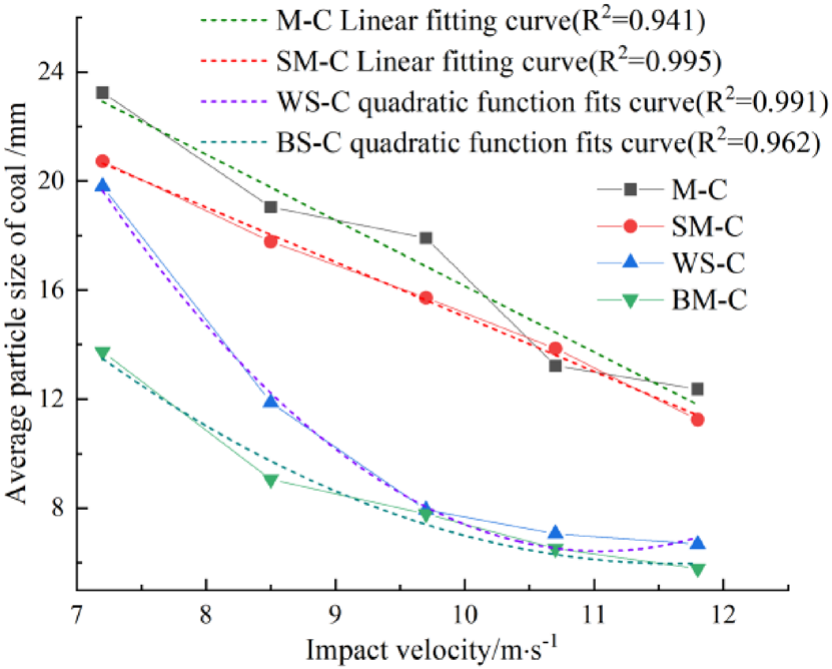
Relationship between average particle size of coal and impact velocity.

From Fig. 12. we can know that the average coal particle size of BS-C specimens was 6.52 mm, showing that the fragment degree of coal sample significantly exceeded that of other groups under the same impact velocity. The same situation was observed in BS-C specimens at other impact velocities. Under the same incident energy, as the strength of the rock increases, the rock plays a more important role in energy accumulation and transmission. The energy absorbed by the CRCB specimen is consumed mainly to initiate and propagation micro-cracks in the coal sample, intensifying its fragmentation. When calculating the fractal dimension, the fractal of the coal sample has a large contribution to the overall fractal of the CRCB specimens, resulting in a large overall fractal dimension of the CRCB specimens.

## Discussion

1. The CRCB specimens can be regarded as two elastic–plastic bodies in series. Under impact load, the coal sample and rock sample in M-C specimens and SM-C specimens have undergone compaction stage, elastic stage, plastic stage and post-peak stage. However, the sandstone sample in WS-C and BS-C specimens may only undergo compaction stage and elastic stage. Due to the different mechanical properties of coal and rocks, the time and duration of coal and rocks entering the deformation and failure stage are different, and the strain response law is also different. In addition, When the cracks tip stress at the interface is greater than the strength of "weakened" sandstone, the dynamic load may not reach the strength of sandstone, but it will also cause the failure of sandstone samples. In this case, the deformation of coal will continue to increase while the elastic deformation of sandstone will suddenly decrease. In summary, the above tow reasons eventually lead to different forms of stress–strain curves of CRCB specimen.
2. After the CRCB specimens failed, part of the broken body at the interface was still in the combined form (Table 3). This implies that the failure process of the CRCB specimens was complicated. Therefore, the interface effects should be considered when analysing the deformation and failure of the combined structure instead of only from the coal or rock mass components.
3. When calculating the fractal dimension of the CRCB specimens, the author did not distinguish the coal and rock components. However, when calculating the average particle size of the coal sample broken blocks, the coal and rock samples were separated and then sieved. Although this method may lead to a certain error from the real value, it was sufficient to reflect the energy dissipation pattern and the crushing characteristics of coal samples.

## Conclusions

The results obtained made it possible to draw the following conclusions:

1. At low impact velocities, the difference in the wave impedance matching greatly impacted the stress wave propagation in the CRCB specimens. However, with the increase of impact velocity, the differential effect of wave impedance gradually weakens.
2. With an increase in impact velocity, the strength and peak strain exhibited obvious strain rate effects, the incidence energy increases linearly while the proportion of reflected energy decreases linearly. The strength and ratio of the absorbed energy changed approximately as a power function. After reaching the specimens strength, the CRCB specimens have strain-hardening characteristic, with the stress–strain curve showing a decrease pattern of "ups and downs".
3. With increasing of rock strength, the coal body is dominated by tensile failure, and the failure mode of rock samples gradually changes from tensile failure to shear failure. The crushing degree of the coal samples in the CRCB specimens gradually intensified with the impact velocity and rock strength. The crushing particle size of coal gradually changed from block to powder, and the fractal dimension of CRCB specimens gradually increased. When the strength of the rock was high, the rock mainly played the role of energy accumulation and transmission, while the coal body was the energy consumed body.
